# Role of Green Macroalgae *Enteromorpha Prolifera* Polyphenols in the Modulation of Gene Expression and Intestinal Microflora Profiles in Type 2 Diabetic Mice

**DOI:** 10.3390/ijms20010025

**Published:** 2018-12-21

**Authors:** Guopeng Lin, Xiaoyan Liu, Xin Yan, Dan Liu, Chengfeng Yang, Bin Liu, Yifan Huang, Chao Zhao

**Affiliations:** 1 College of Food Science, Fujian Agriculture and Forestry University, Fuzhou 350002, China; tkmcrxwn@gmail.com (G.L.); yanxin18ch@gmail.com (X.Y.); LiuDan379@163.com (D.L.); binliu618@163.com (B.L.); 2 College of Food Science and Nutritional Engineering, China Agricultural University, Beijing 100083, China; liuxiaoyan8112@163.com (X.L.); cfyang07@cau.edu.cn (C.Y.); 3 Fujian Provincial Key Laboratory of Veterinary Medicine and Animal Health, Fujian Agriculture and Forestry University, Fuzhou 350002, China; zjhyfang@163.com; 4 Fujian Province Key Laboratory for the Development of Bioactive Material from Marine Algae, Quanzhou Normal University, Quanzhou 362000, China

**Keywords:** *Enteromorpha prolifera*, polyphenols, antidiabetic activity, signaling pathway, intestinal microflora

## Abstract

Effects of green macroalgae 55% ethanolic extract *Enteromorpha prolifera* through an ultrafiltration membrane of 3 kDa (EPE3k) on antidiabetic activity, gut microbiota, and regulation mechanism were investigated in high-fat/high-sucrose diet and streptozocin-induced diabetic mice. The structural characterizations of its major compounds in EPE3k were determined by ultra-performance liquid chromatography-quadrupole/time of flight mass spectrometry. Furthermore, the intestinal microflora modulation in diabetic mice was also investigated with high-throughput 16S rRNA gene sequencing. The proposed presence of polyphenols in EPE3k was confirmed. EPE3k could significantly decrease the fasting blood glucose and improve fasting glucose tolerance. The hypoglycemic effect of EPE3k was via activation of phosphatidylinositol 3-kinase and suppression of c-Jun N-terminal kinase in liver. EPE3k treatment significantly increased the relative abundance of *Akkermansia* and decreased the proportion of *Alistipes* and *Turicibacter*. The above results indicated that EPE3k could be provided as a new potential therapy for the treatment of type 2 diabetic mellitus.

## 1. Introduction

Type 2 diabetes mellitus (T2DM) is the most common form of diabetes, accounting for more than 90% of all diabetes. Asia is one major area of the rapidly emerging T2DM global epidemic, particularly China and India [1]. Insulin resistance and pancreatic β-cell dysfunction are the two features of T2DM. The hypoglycemic drugs in the market mainly improve insulin resistance and increase insulin production, but most of them have different degrees of side effects for certain symptoms and reactions [2]. Therefore, the novel natural medicines with high efficiency and low toxicity have a critical preparedness for against T2DM [3]. In particular, marine algae are well-known healthy food and contains a variety of biologically active compounds such as pigments, fucoidans, phycocolloids, and phenolic compounds [2,4]. Marine algal polyphenols had a variety of physiological activities such as hypoglycemic, hypolipidemic, anticoagulation, antitumor, antivirus, and anti-aging [5,6,7,8]. *Enteromorpha prolifera* is one of the most popular marine green algae in Asian countries and contains many bioactive compounds [9]. The previous studies have indicated that *E. prolifera* polysaccharides attenuated non-alcoholic fatty liver disease in high-fat diet rats. It was increasingly recognized that these natural phytochemicals contributed to the functional nutritional food industries [10,11]. However, functional ingredients of *E. prolifera* have been rarely found to possess antidiabetic properties.

Recent studies have shown that insulin stimulation, including target tissue uptake and utilization of glucose, is accomplished through a series of signal transduction processes, such as phosphatidylinositol 3-hydroxy kinase (*PI3K*) and c-Jun N-terminal protein kinases (*JNK*). Any impairment or obstacle in these signal transduction process can lead to insulin resistance [12,13,14,15,16]. *PI3K* is recognized as one major gene associated with the development of insulin resistance [17]. Moreover, *JNK* also provides a target for the treatment of T2DM [14]. The gut microbiota has played an important role in the regulation of host metabolism, maturation of the immune system, and development of metabolic diseases. In addition, gut microbiome disorder can also result in the occurrence and development of T2DM by changing the metabolism of short-chain fatty acids or bile acids. Previous studies have shown that the intestinal microflora composition was associated with the development of T2DM, which has been gradually recognized as a potential diagnostic and therapeutic target for the disease [18].

Functional compounds from marine macroalgae can effectively regulate the balance of gut microbiome and improve glucose metabolism [4]. However, previous investigations on green macroalgae *E. prolifera*, especially low molecular weight compounds, have not yet been reported. In this study, *E. prolifera* were extracted with 55% ethanol and then passed through an ultrafiltration membrane of 3 kDa to obtain the target sample (EPE3k). The potential antidiabetic properties and its mechanism of EPE3k in vivo were investigated. What′s more, the structure and function of intestinal flora were also analyzed by high-throughput sequencing. It’s the first report to study the hypoglycemic effect of *E. prolifera* polyphenols in type 2 diabetic mice.

## 2. Results

### 2.1. Characterization of Potent Major Compounds

Phytochemical analysis of EPE3k resulted in the isolation of four major components (Figure S1). Further chromatographic peaks were observed at different retention times which attempted to unequivocally identify these constituents by quadrupole/time of flight mass spectrometry (QTOF/MS/MS) (Figure S2). The analysis of MS confirmed the proposed presence of four polyphenols (Table 1). Their structures were identified by comparing their MS spectral data with those reported in the literature [19,20]. The partial fragment ions at *m*/*z* were in agreement with previously reported data.

### 2.2. Effects of EPE3k on Fasting Blood Glucose (FBG) Levels and Oral Glucose Tolerance Test (OGTT)

**Figure 1:**
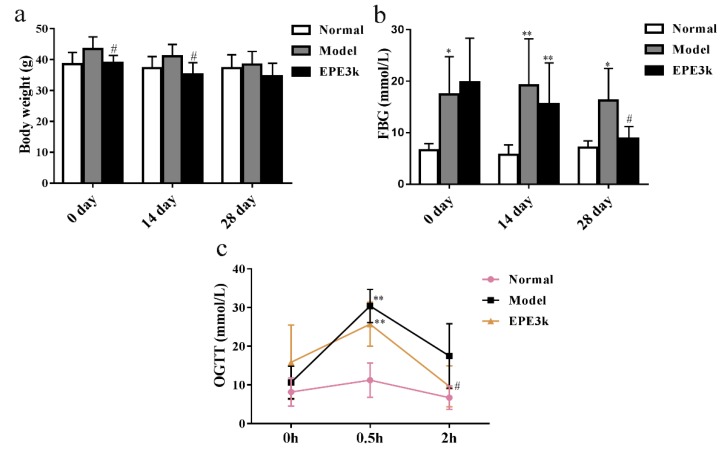
The variation on body weight (**a**), fasting blood glucose (FBG) (**b**), and oral glucose tolerance test (OGTT) (**c**) of type 2 diabetic mice after treated by EPE3k. * *p* < 0.05, ** *p* < 0.01: compared with normal group; **#** *p* < 0.05: compared with model group.

On the 0 and 14 days, body weights of mice treated with EPE3k were significantly lower compared with the model group (Figure 1a). Significant difference in FBG levels was also found between normal group and other two groups at the early experiment (Figure 1b). After four weeks treatment, the FBG levels of mice in EPE3k-treated group were significantly lower than that of model diabetic group. Moreover, it was similar to those in the normal group after 28 days of treatment. The glucose tolerance of mice in model group and EPE3k group had been severely increased, while the blood glucose in those animals was obviously higher than normal group at 0.5 h (Figure 1c). However, after 2 h, lower blood glucose levels were observed in model group and EPE3k treatment group. Furthermore, blood glucose levels in EPE3k group were significantly lower than the model group.

### 2.3. Effect of EPE3k on the Histopathology of Liver in Diabetic Mice

**Figure 2:**
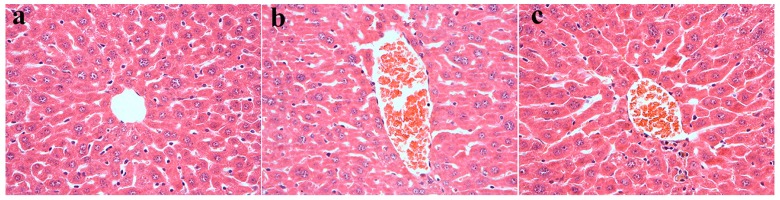
Histopathological analysis of EPE3k on hepatic tissues in the normal (**a**), model (**b**), and EPE3k (**c**) groups with hematoxylin and eosin staining at 400× magnification.

The liver cells of normal group were closely arranged in orderly, and the structure of hepatic lobules was normal (Figure 2a). Compared with normal group, liver cells of model group had a disorder of hepatic cord structure and showed obviously mild inflammation (Figure 2b). The high-sucrose/high-fat diet and streptozocin were harmful to the hepatic tissues of mice. The hepatic cord arrangement in EPE3k-treated group was fuzzy compared to model group, but there was a slight improvement of the degree of inflammation. EPE3k treatment could effectively improve the inflammation of hepatocytes caused by diabetes (Figure 2c). EPE3k had a certain degree of repair function and improved the quality of liver cells in T2DM mice.

### 2.4. Effect of EPE3k on Gene Expression of Signal Pathway in Liver

**Figure 3:**
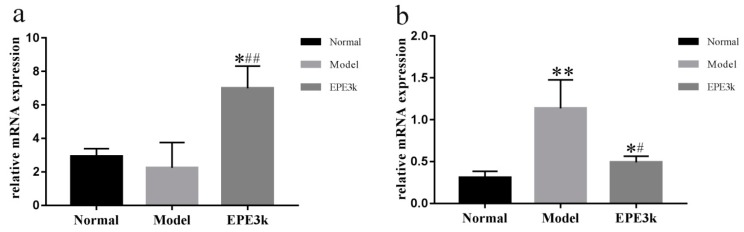
Effect of EPE3k on mRNA expression of *PI3K* (**a**) and *JNK1* (**b**) in liver of type 2 diabetic mice. * *p* < 0.05 and ** *p* < 0.01; compared with normal group; # *p* < 0.05 and ## *p* < 0.01, compared with model group.

After four weeks of experiment, the mRNA expression of *PI3K* in the liver of EPE3k group was significantly higher than that of model group, while the expression in model group was lower than normal group (Figure 3a). Moreover, there was a significant increase in the mRNA expression level of *JNK1* gene in diabetic mice compared with normal mice. The mRNA expression of *JNK1* in mice of EPE3k group was significantly lower than that in the model group and closed to that in the normal group (Figure 3b). Meanwhile, the protein expressions of *PI3K* in EPE3k group was also increased apparently compared with model group, and treatment of EPE3k could significantly inhibit the expression of *JNK1* (Figure S3).

### 2.5. Effect of EPE3k on the Intestinal Microflora

**Figure 4:**
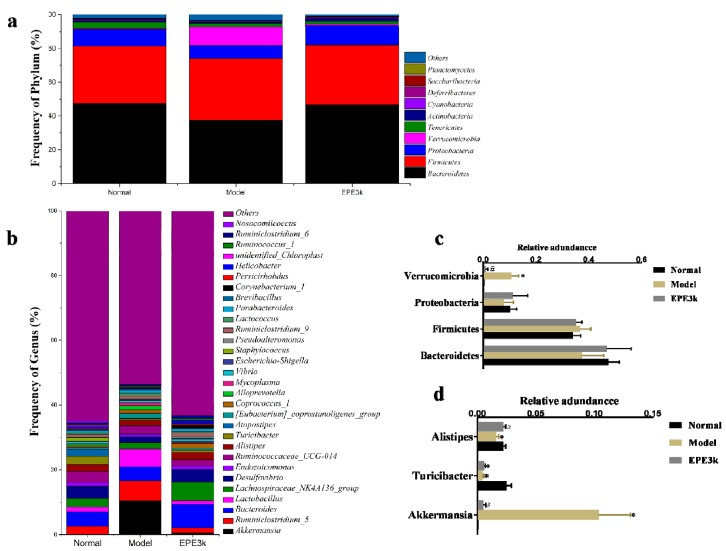
Effects of EPE3k treatment on the relative abundances of intestinal microflora at phyla (**a**) and genus (**b**) levels. The largest phyla (**c**) and genus (**d**) levels changes in microbiota were shown in different groups. * *p* < 0.05: compared with normal group; **#** *p* < 0.05: compared with model group.

The abundance of intestinal bacteria in each group at the phylum level was determined. The results showed that the intestinal bacteria identified were mainly Bacteroidetes, Firmicutes, Proteobacteria, Proteobacteria, Verrucomicrobia, Tenericutes, and Actinobacteria (Figure 4a). Furthermore, Bacteroidetes, Firmicutes, and Proteobacteria were the dominant bacteria detected in the intestinal contents of all the mice at phylum level (Figure 4c). The proportion of Verrucomicrobia was increased significantly in the model group compared with normal group, but it was decreased in EPE3k group while compared with model group. It has documented that *Akkermansia* in EPE3k-treated group also significantly decreased compared with model group (Figure 4b,d). Besides, the proportion of *Turcibacter* bacteria was decreased significantly in both model and EPE3k groups. On the contrary, the proportion of *Alistipes* bacteria in EPE3k group increased significantly compared with the model group.

### 2.6. Correlation between Biological Indexes, Gene Expression Levels and Cecal Microbiota

**Figure 5:**
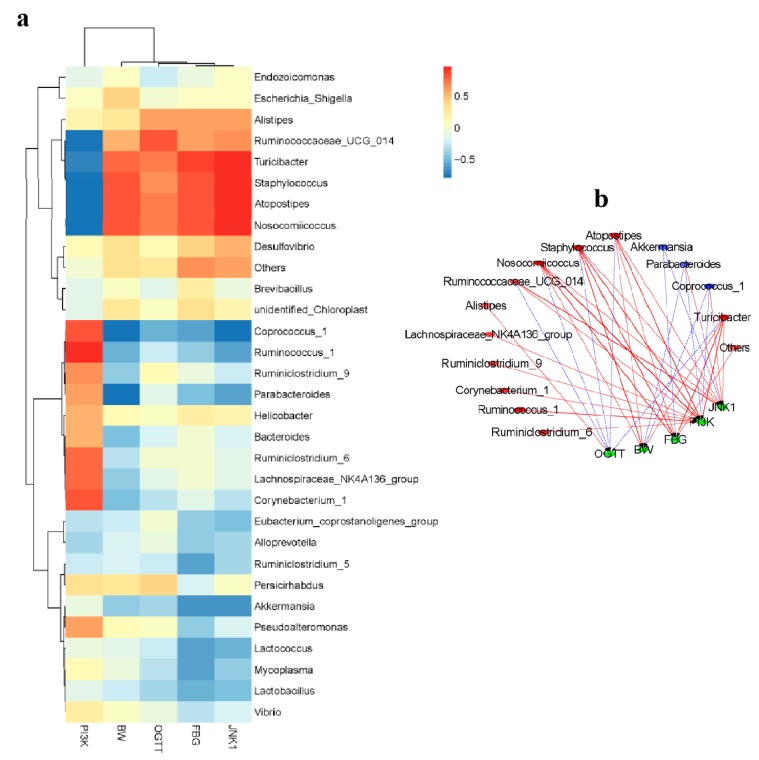
(**a**) Analysis of heat map of gut microbiota with biochemical indexes and mRNA expression levels. Red region represents positive correlation, blue region represents negative correlation, and the shades of color indicates the strength of relevance among them. (**b**) Analysis of network of gut microbiota, biochemical indexes, and gene expression levels. The solid red line and dotted blue line represented positive and negative correlation, respectively. In addition, the line width indicated the strength of correlation; the edges were drawn in the network using the Spearman correlation (|*r*| > 0.6).

All samples were selected for the top 30 genera of species abundance at the genus level, and the intestinal flora of type 2 diabetes was clustered from the T2DM mice (Figure 5a). A correlation index (Pearson) was calculated for the biochemical indicators, gene expression levels, and gut microbiota of all samples, then a table of species correlation coefficients plotted as heat map was obtained. Among the top 30 genera of species, the 15 genus with the highest Pearson indexes were selected (Figure 5b). *Akkermansia* was negatively correlated with FBG and *JNK1*. *Parabacteroides* and *Coprococcus_1* were also positively correlated both *PI3K* and BW. Not only other gut flora was positively correlated with FBG, but also *Ruminiclostridium_6*, *Ruminococcus_1*, *Corynebacterium_1*, *Ruminiclostridium_9*, and *Lachnospiraceae_NK4A136_group* all were positively correlated with *PI3K*.

## 3. Discussion

In this study, assessment of anti-diabetic potential of the phytochemicals from *E. prolifera* 55% ethanol extract filtrated with a 3-kDa ultrafiltration membrane in mice was determined. The maintained weight in type 2 diabetic mice has been measured. The fasting blood glucose level of the diabetic model group was significantly higher than that of the normal group from the beginning to 28 days, indicating that the diabetic mice models were successfully induced. At all stages, EPE3k-treated group has an obviously hypoglycemic effect on type 2 diabetic mice. After two h, the blood glucose with EPE3k treatment decreased significantly compared with model group. Hematoxylin and eosin staining results showed that the liver tissue cells of normal group were arranged tightly, while the structure and arrangement of the hepatic lobule were normal. Compared with normal group, the liver cord structure in the model group was disordered and the liver was fine cells show marked mild inflammation. EPE3k group had some repair function for liver in the degree compared with type 2 diabetic mice. The present results suggested that EPE3k treatment had a strong hypoglycemic activity and improved the oral glucose tolerance on streptozocin-induced diabetic mice. Some interesting initial results on glucose metabolism and *PI3K*/*Akt* signaling pathway were also explained. It was similar to the previous studies which indicated that *Caulerpa lentillifera* extract regulated glucose uptake and homeostasis via *PI3K*/*Akt* pathway in myocytes and db/db mice [21]. In general, the results suggested that EPE3k treatment had marked hypoglycemic activity on streptozocin-induced diabetic mice fed with high sucrose and high fat diets.

Glucose is used in the target tissues by two major pathways including *PI3K* [18]. Glucose homeostasis is broken by the absence of the *PI3K*/*Akt* pathway in insulin-sensitive tissues, leading to the accumulation of glucose in the blood. In the insulin target cells, serine and threonine residue sites of IRS1 and IRS2 are phosphorylated by *JNK1*/2, which resulted in a decrease in tyrosine phosphorylation level of the former and a decrease in *PI3K* signal transduction [19]. Both of them could ultimately lead to T2DM. The liver tissues of the experimental mice were detected by real-time PCR. In order to investigate whether EPE3k treatment could effectively improve the gene expressions related to a hypoglycemic pathway in T2DM. The mRNA and protein expressions of *PI3K* and *JNK1* genes were determined. EPE3k treatment could improve the key *PI3K* and partly inhibit the *JNK* signal pathway by promoting glucose uptake in the peripheral tissue. The above results indicated that EPE3k improved the liver insulin resistance in diabetic mice by regulating *JNK1* and *PI3K* signaling pathway.

The natural active substances can be utilized in the intestinal tract after consumed by human body, and then regulated the species to restore metabolic behavior and hopefully achieve the purpose of treatment [22]. The intestinal flora was mainly composed of nine phylum bacteria including, Bacteroidetes, Firmicutes, Proteobacteria, Actinobacteria, VadinBE97, Fusobacteria, Verrucomicrobia, Cyanobacteria, and Spirochaeates, of which Bacteroides and Firmicutes were the absolute advantages (>90%). There were significant differences in gut bacteria between type 2 diabetic patients and healthy people at the phyla and genus levels [23,24]. The relative abundance of scleredema crassipes in diabetic patients was higher. And Bacteroides and Proteus were lower compared with normal people based on the previous reports, which showed that microorganisms residing in the intestinal tract participated in the metabolic process of various nutrients in the host body [25]. The abundant changes of the principal bacterial phylum, most notably within the Bacteroidetes, Firmicutes, Proteobacteria, Actinobacteria, and Deferribacteres phylum, were observed in treatment and diabetic mice in this study. The results in the experiment showed that, at the phylum level, the Bacteroides in model group was lower than that of normal group, while the Firmicutes in model group was higher. In the EPE3k treatment group, Bacteroides was up-regulated and Firmicutes was down-regulated. Firmicutes promoted the caloric absorption from diet and the fat storage in intestinal cells. At the genus level, EPE3k treatment could significantly regulate the abundance of *Akkermansia*, *Alistipes,* and *Turicibacter*, and tended to be a positive development of flora structure in mice. These results seemingly corresponded with the histological characteristics of the liver, attenuated levels of blood glucose, and decreased body weight of EPE3k-treated mice. It’s confirmed that gut microbiota was involved in the regulation of T2DM through altering the secretion of incretins. Thus, EPE3k has also potential applications as probiotic treatments for T2DM.

## 4. Materials and Methods

### 4.1. Preparation of Ethanolic Extract from E. prolifera

The air-dried powder of *E. prolifera* which attained from Lanbao Marine Bio-technology Co., Ltd (Qingdao, China), was extracted with 55% ethanol by ultrasound-assisted extraction (60 °C, 45 kHz) for 90 min. Then the extract was concentrated and passed through 3 kDa ultrafiltration membrane freeze dried.

### 4.2. Ultra-Performance Liquid Chromatography-Mass Spectrometric (UPLC-MS) Analysis

After centrifuging at 12,000 rpm for 10 min at 4 °C, the extract was filtered through Syringe filters (PTFE, 0.22 μm pore and 13 mm diameter, Millipore Millex, Billerica, MA, USA) and then injected to UPLC system (Waters Co., Milford, MA, USA). Chromatographic separations were carried out on a Waters Acquity UPLC I-Class with C18 column (1.8 μm, 2.1*100 mm, Waters Co.). Water with 0.1% (*v*/*v*) formic acid (solvent A) and acetonitrile with 0.1% (*v*/*v*) formic acid (solvent B) were used as the mobile phase. The UPLC elution condition was optimized as follows: 99% of solvent A (0–0.25 min), 99–1% of solvent A (0.25–16.25 min), 1% of solvent A (16.25–17.00 min), 1–99% of solvent A (17.00–17.01 min), and 99% of solvent A (17.01–20.00 min) at a flow rate of 0.45 mL/min [16,18]. The injection volume was 1 μL aliquot of each sample. SYNAPT G2-Si HDMS (Waters Co., Milford, MA, USA) equipped with an electrospray ion source (ESI) was used. The MS parameters were set as follows: the scan range from 50–1200 *m*/*z*, scan time of 0.2 s, source offset of 80, nebulizer gas flow (Bar) of 6.5, capillary voltage of 2.0 kV at ESI^+^, ion source temperature of 120 °C, desolvation temperature of 450 °C, and desolvation gas flow rate of 800 L/h. The data acquisition mode was set as MSE with the low energy of 4 eV and elevated energy ramping from 10–60 eV to acquire the protonated or deprotonated molecule, in which MS/MS fragmentation was performed with collision-induced dissociation using collision energy from 10 to 60 eV. The diode array detector was set to a wavelength of 190–700 nm. The mass spectrometer and UPLC system were controlled by MassLynx 4.1 software (Waters, Milford, MA, USA).

### 4.3. Animals Experiments

ICR male mice (Specific Pathogen Free, 8-week-old; 18–22 g) were purchased from Fuzhou General Hospital of Nanjing Military Region (Fuzhou, China). All experimental protocols were in accordance with the guidelines of laboratory animal welfare ethics and daily animal care guidelines, which were approved by the board of Fuzhou General Hospital of Nanjing Military Region (27/03/2016 & FZZY-2016). After one week, ten mice in the experimental group were randomly chosen as normal group fed with standard chow, and the other 20 mice were fed a high-sucrose/high-fat diet (15% lard, 15% sucrose, 1% cholesterol, 10% yolk, 0.2% sodium deoxycholate, and 58.8% standard chow) to induce diabetes. After 4 weeks, all mice fasted for 12 h with free access to drinking water, then mice were intraperitoneally injected with fresh streptozocin solution (dissolved in 0.1 mol/L citrate buffer, pH 4.5) at a dose of 45 mg/kg body weight. Mice in the normal group were injected with citrate buffer. All animals were injected three times per day. The FBG levels were detected by OMRON glucose meter (Kyoto, Japan) at 24 h since the last injection. Mice with FBG levels over 11.1 mmol/L were regarded as type 2 diabetic mice. Type 2 diabetic mice were randomly divided into two groups as model and EPE3k treatment groups (300 mg/kg body weight). Mice were gavaged with water daily.

### 4.4. Blood Sample Collection and Oral Glucose Tolerance Test

Body weight and FBG were measured every two weeks. After 4 weeks of continuous intragastric oral administration, all mice were fasted for 12 h with free access to drinking water for OGTT. After administrated orally with glucose of 2 g/kg, the blood glucoses were tested at 0, 0.5, and 2 h. Mice were sacrificed by cervical dislocation, and then the liver and cecum were dissected and washed with saline. Part of the liver was fixed with 10% formaldehyde solution and the rests stored at −80 °C. Cecum was directly collected and stored immediately by drikold and kept at −80 °C for further use.

### 4.5. Histopathological Analysis

The liver was fixed in 10% of formalin. After embedding in paraffin, these organs were cut into 2 mm sections and stained with hematoxylin and eosin. The sections were examined under a light microscope.

### 4.6. Dynamic Profile of Intestinal Microflora in Response to EPE3k

The bacterial genome DNA was extracted from mice intestinal contents by using E.Z.N.A.TM Mag-Bind Stool DNA Kit (Omega, Norcross, GA, USA) according to the guideline of manufacturer. The V3-V4 regions of the 16S rRNA genes were amplified with the designed primer pairs (F, 5′-CCTACGGGNGGCWGCAG-3′ and R, 5′-GACTACHGGTATCTAATCC-3′). The mixture contained 10–20 ng of DNA template, 2× Taq master Mix (Thermo Scientific, Waltham, MA, USA), and 10 μM of primers F/R were used for PCR reactions. The thermal cycling conditions were used as follows, initial denaturation at 94 °C for 3 min; 5 cycles at 94 °C for 30 s, 45 °C for 20 s, and 60 °C for 30 s; 20 cycles at 94 °C for 30 s, 55 °C for 20 s, and 72 °C for 30 s; and a final extension at 72 °C for 5 min. Amplicons were purified and quantified by using the Agencourt AMPure XP (Beckman, CA, USA) and Qubit dsDNA HS Assay Kit (Invitrogen, NY, USA), respectively, and then sequenced on the Illumina Miseq™ platform (San Diego, CA, USA). The bacterial sequences were assigned taxonomically based on the Ribosomal Database Project classifiers. The microbial community structures in different samples were compared using FastUniFrac (http://bmf2.colorado.edu/fastunifrac/) on the basis of the phylogenetic relationships.

### 4.7. Real-Time PCR

Using Trizol reagent (Invitrogen, NY, USA) to get total RNA from the liver samples of mice, 1st-stand cDNA was synthesized with cDNA synthesis kit (Takara, Dalian, China). RT-PCR of *β-actin*, *PI3K*, and *JNK1* were performed using SYBR Green RT-PCR kit (Invitrogen, NY, USA). Their specific primers were listed as follows: *β-actin*, F: 5′-ACATCCGTAAAGACCTCTATGCC-3′, R: 5′-TACTCCTGCTTGCTGATCCAC; *PI3K*, F: 5′-CCAAATGAAAAGAACGGCTA-3′, R: 5′-GCGACTTCAGCTTATCATGG-3′; *JNK1*, F: 5′-CAGAAGCAAACGTGACAAC-3′, R: 5′-AAGAATGGCATCATAAGCTG-3′. Amplifications were performed by the following conditions: 95 °C 10 min for initial denaturation, 95 °C for 15 s, 40 cycles at 60 °C for 50 s. ABI StepOne plus Real-time PCR system (Applied Biosystems, Foster City, CA, USA) was used to analyze the relative quantitative of mRNA, which was normalized to that of *β-actin*.

### 4.8. Protein Extraction and Western Blotting

The livers of mice were homogenized with SDS lysis buffer and centrifuged at 8000 rpm for 8 min at 4 °C. The proteins were segregated by 10% SDS-PAGE and then were transferred to PVDF membranes (Sangon, Shanghai, China). The membranes were blocked with QuickBlock™ Blocking Buffer for 1 h at 4 °C, then were incubated for 48 h at room temperature using antibodies against *PI3K*, *JNK1*, and *β-actin* (1:1000; Beyotime Biotechnology, Shanghai, China) [18].

### 4.9. Statistical Analysis

All values were expressed as mean ± SD. Statistical significance was determined using analysis of variance (ANOVA) followed by Duncan test. A *p*-value of less than 0.05 was considered statistically significant.

## 5. Conclusions

In this study, the anti-diabetic effects of *E. prolifera* polyphenols have been validated in vivo. Effective treatment was achieved at intestinal microflora. Intriguingly, among them, Bacteroidetes increased and Firmicutes decreased. Specifically, the abundance of beneficial bacteria such as *Akkermansia*, *Alistipes,* and *Turicibacter* in intactness could be enriched at the genus level. It has been observed that EPE3k could also be adjusted by up-regulating *PI3K* expression level and inhibition of *JNK1* activity by reducing the concentration of blood glucose. This study verified the prevention and treatment of T2DM of *E. prolifera* polyphenols by classical animal experiments, biochemical analysis, histomorphometry, and associated with an angle of the intestinal tract.

## Figures and Tables

**Table 1:** Identification of EPE3k by UPLC-QTOF-MS/MS.

Peak No.	RT (min)	[M + H]^+^	Fragment Ions	Formula	Identified Compounds	Ref.
1	0.65	277	277[M + H]^+^, 235[M + H-C_3_H_6_]^+^, 151[M + H-C_5_H_14_O]^+^, 133[M + H-C_8_H_16_O_2_	C_18_H_28_O_2_	Estr-5(10)-ene-3,17-diol	[19]
2	4.99	179	179[M + H]^+^,161[M + H-H_2_O]^+^, 133[M + H-H_2_O-CO]^+^, 105[M + H-H_2_O-2CO]^+^	C_10_H_10_O_3_	Regiolone	[19]
3	5.64	449	449[M + H]^+^, 299[M + H-Glc]^+^	C_21_H_20_O_11_	Luteolin-6-C-glucoside	[20]
4	6.59	597	399[M + H-Orha-2H_2_O]^+^, 135[M-H-Rha-Glc-C_7_H_4_O_4_]^−^, 107[M-H-Rha-Glc-C_8_H_8_O_2_-CO_2_]^−^	C_27_H_32_O_15_	Neoeriocitrin	[20]

## Supplementary Materials

Supplementary materials can be found at http://www.mdpi.com/1422-0067/20/1/25/s1.
