# Supplementary material for: Role of Green Macroalgae Enteromorpha Prolifera Polyphenols in the Modulation of Gene Expression and Intestinal Microflora Profiles in Type 2 Diabetic Mice
Source: Int J Mol Sci. 2018 Dec 21;20(1):25. doi: 10.3390/ijms20010025 (PMC6337142; doi:10.3390/ijms20010025)
Supplement: Supplementary file 1 [file ijms-20-00025-s001.pdf]

Supplementary Materials

Role of green macroalgae *Enteromorpha prolifera* polyphenols in the modulation of gene expression and intestinal microflora profiles in type 2 diabetic mice

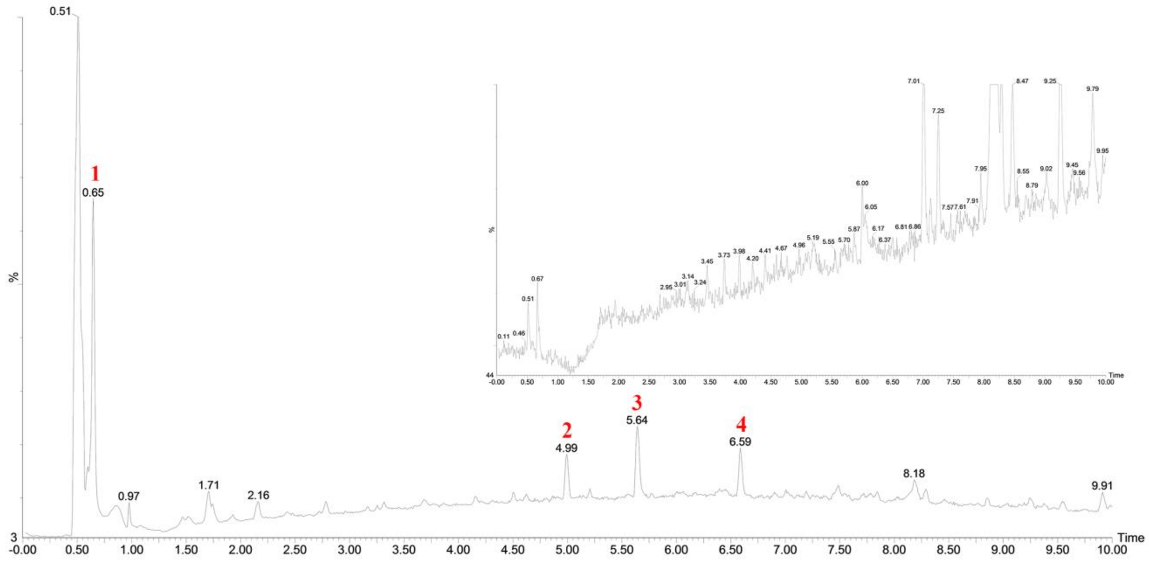

Figure S1. Chromatographic peaks of EPE3k in UPLC. The graphic in the top right was presented the spectrums of the blank.

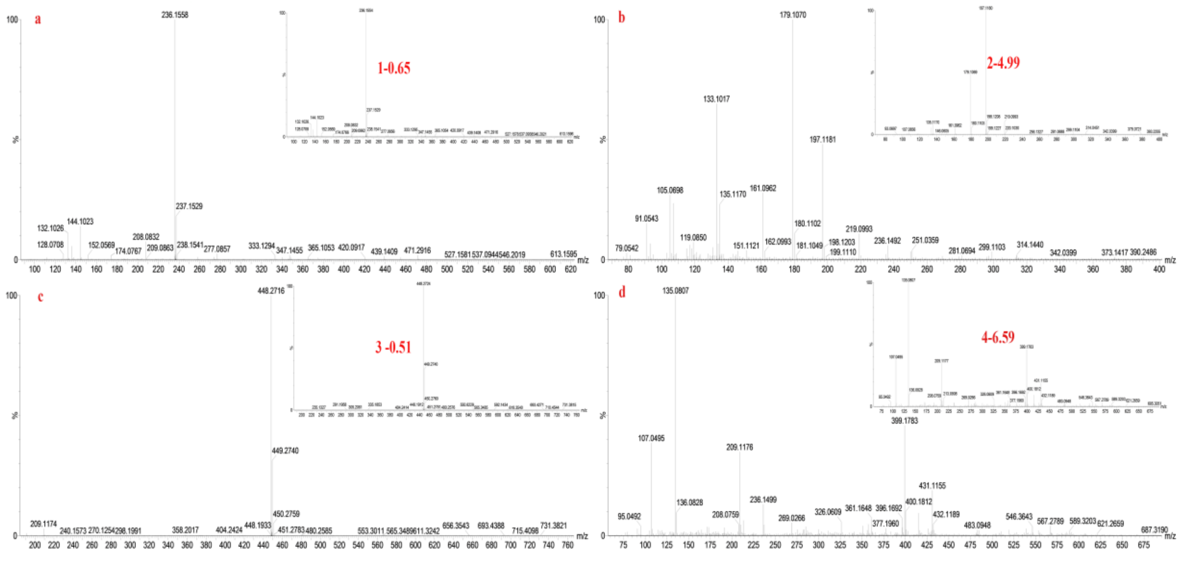

Figure S2. Representative chromatographs of EPE3k with UPLC-Q-TOF-MS/MS.

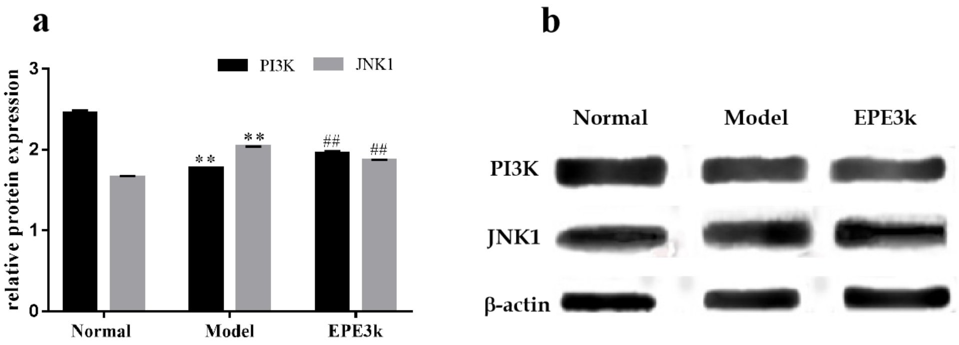

Figure S3. Effects of EPE3k treatment on the protein expression levels of PI3K and JNK1 (**a**), and western blot analysis (**b**). Signals were normalized with those of β-actin. The values were expressed as mean ± SD ($n \geq 3$). ## $p < 0.01$, compared with model group; ** $p < 0.01$, compared with normal group.
